# Improved Quality of Life in Children and Families Following Enrollment in a Pediatric Palliative Care Program: A Prospective Cohort Study

**DOI:** 10.3390/children13020196

**Published:** 2026-01-30

**Authors:** Jéssica H. Guadarrama-Orozco, María G. Mendoza-Martínez, Sergio E. Bautista-Téllez, Paola Yañez-Maldonado, Karina Mendoza-de la Vega, María F. Castilla-Peon

**Affiliations:** 1 Hospital Infantil de México Federico Gómez, Palliative Care Service, Dr. Márquez, 162, Doctores, Mexico City 06720, Mexico; jessypedia@gmail.com (J.H.G.-O.); karina.mendoza.delavega@hotmail.com (K.M.-d.l.V.); 2 Facultad de Medicina, Universidad Nacional Autónoma de México, Escolar 411, Copilco Universidad, Mexico City 04510, Mexico; sergioedgarbautistatellez999@gmail.com (S.E.B.-T.); paolayanezmaldonado@gmail.com (P.Y.-M.); 3 Hospital Psiquiátrico Infantil Dr. Juan N. Navarro, Comisión Nacional de Salud Mental y Adicciones, San Buenaventura 86, Belisario Domínguez, Mexico City 01480, Mexico

**Keywords:** palliative care, quality of life, pediatrics, family, Mexico, cohort studies

## Abstract

**Highlights:**

- This study demonstrates significant improvements in the quality of life of both children and their families following enrollment in a pediatric palliative care program at a public tertiary hospital in Mexico City.
- Families living outside the Mexico City metropolitan area reported lower baseline quality of life but experienced greater improvement over time.
- Pediatric palliative care services can benefit families across a range of clinical and social contexts, including those with limited access to local care.
- These findings support the expansion of pediatric palliative care programs in resource-constrained settings.

**Abstract:**

**Background/Objectives**: Pediatric palliative care seeks to relieve suffering and improve the quality of life of children with severe conditions and their families. This prospective cohort study assessed changes in quality of life following enrollment in a pediatric palliative care program at a tertiary care center in Mexico and explored factors associated with these changes. **Methods**: Children with life-limiting or severe disabling conditions were followed at baseline, 3 months, and 6 months. Quality of life was measured using the Pediatric Quality of Life Inventory (PedsQL™) Cancer Module for oncologic patients and the PedsQL™ Family Impact Module for all families. **Results**: A total of 166 families completed the Family Impact Module questionnaires, and 116 oncologic patients completed the Cancer Module. Mean children’s PedsQL Cancer Module scores improved from 58.9 to 77.9, and family scores improved from 60.1 to 78.8 over six months (both *p* < 0.001). Families of oncologic patients and those residing outside the Mexico City metropolitan area had lower baseline scores (adjusted differences −9.84, 95% CI: −15.9 to −3.77; and −6.9, 95% CI: −12.38 to −1.44, respectively); however, the latter group showed a greater rate of improvement over time, contrary to our initial hypothesis—survival varied by diagnosis, with longer survival observed in children with neurologic or intracranial conditions. **Conclusions**: The quality of life of families and pediatric oncologic patients showed improvement over time following enrollment in a specialist pediatric palliative care program in a middle-income setting. Equitable access should be ensured for families affected by chronic conditions, particularly those living beyond major urban areas.

## 1. Introduction

Pediatric palliative care is a multidisciplinary approach aimed at alleviating the physical, psychological, and social suffering of children with life-limiting conditions and supporting their families [1]. While pediatric palliative care is well established in high-income countries, services in low- and middle-income settings remain underdeveloped [2].

In Mexico, leading causes of pediatric mortality include cancer, neurological disorders, and congenital anomalies, with respective rates of 5.0, 3.8, and 3.7 deaths per 100,000 children [3]. Despite this burden, few hospitals offer structured pediatric palliative care programs. Evidence on the benefits of pediatric palliative care for patients and families in low- and middle-income settings remains limited, underscoring the need for longitudinal assessments of quality of life in these populations. Families in these contexts often face additional socioeconomic and logistical stressors that worsen their quality of life.

Although most pediatric palliative care models are cancer-focused, children with other chronic, disabling conditions may have prolonged disease courses and complex care needs that warrant tailored palliative support.

Despite the recognized benefits of pediatric palliative care, empirical descriptions of quality-of-life trajectories in children who do not receive such care are extremely limited. Longitudinal comparative studies are scarce, and studies designed to follow pediatric patients with life-limiting conditions without palliative intervention are not ethically feasible. Systematic reviews consistently highlight the lack of longitudinal data and controlled comparisons in pediatric palliative care research [4,5,6]. Evidence from randomized trials and controlled studies suggests that, in the absence of specialized palliative interventions, quality-of-life outcomes in control groups tend to remain stable or fail to improve over time [7,8,9].

In middle-income countries such as Mexico, where access to specialized pediatric palliative care is heterogeneous and strongly influenced by geographic and socioeconomic factors, families may experience additional burdens related to travel, fragmented care, and limited community-based support. These challenges are particularly relevant for families living outside large metropolitan areas, where the absence of structured palliative care may disproportionately affect both children’s and caregivers’ quality of life.

This study aimed to evaluate the quality of life trajectories of patients and their families enrolled in a pediatric palliative care program. Demographic and clinical factors influencing changes in quality of life over time were also examined. We hypothesized that quality of life would worsen over time and that families living outside the Mexico City metropolitan area would experience less improvement after enrolling in the program compared to those residing within the city.

## 2. Materials and Methods

This prospective cohort study was conducted at the Hospital Infantil de México Federico Gómez, a tertiary referral center in Mexico City. Children aged 0–18 years with life-limiting or severe disabling conditions were referred to the pediatric palliative care Unit by their treating physicians. The unit offers integrated medical, psychological, and social care, along with home visits and telephone follow-up. All families receive telephone follow-up, caregiver training for home-based care, and coordination with local health services as needed. Families residing within the Mexico City metropolitan area additionally receive regular home visits from the palliative care team and have access to medical equipment loans when required, whereas families living outside the metropolitan area do not.

Upon enrollment, each family underwent a multidimensional assessment conducted by a pediatrician, psychologist, and social worker. Individualized care plans were developed focusing on symptom control, caregiver education, and coordination with local health services. Baseline information and demographic data were collected at the hospital for every new patient enrolled between 1 July 2021 and 28 February 2025. Follow-up telephone interviews were conducted at 3 and 6 months to assess the quality of life of both patients and their families.

Given the nature of pediatric palliative care populations, follow-up completeness was inherently influenced by disease severity and end-of-life processes, with attrition driven predominantly by mortality. Participant flow diagrams for the full cohort and the oncologic sub-cohort are provided in Figures S1 and S2.

Instruments: Quality of life was assessed using validated Spanish-language instruments: the PedsQL™ 3.0 Cancer Module for children aged 2–18 with cancer, and the PedsQL™ 2.0 Family Impact Module for caregivers of all patients [10,11,12,13,14,15]. Both use a 0–100 scale, with higher scores reflecting better quality of life. Socioeconomic status was classified using the Mexican Association of Market and Opinion Intelligence (AMAI) index, which categorizes households from D (lowest) to AB (highest) based on education, housing, and access to resources [16].

Analysis: Participants with more than 20% unanswered questionnaire items were excluded. Descriptive analyses were performed for baseline characteristics. Simple linear regression models were applied to examine associations between demographic and clinical factors and baseline quality-of-life scores, with coefficients reported to reflect the magnitude of differences between comparison groups.

To evaluate longitudinal changes in quality-of-life scores, linear mixed-effects models were fitted. Measurement timepoint (baseline, 3 months, and 6 months) and relevant covariates were included as fixed effects, along with interaction terms to assess whether trajectories differed by key factors. Participant-level random effects were specified using an unstructured covariance matrix. Models were estimated using maximum likelihood. Separate models were specified for each factor potentially associated with quality of life and its rate of change, including age group, sex, diagnostic category (oncologic, neurologic, and other), place of residence (Mexico City metropolitan area vs. non-metropolitan), socioeconomic status (AMAI D vs. higher), and survival time from baseline assessment. Survival time was included as a proxy for clinical status, assuming shorter survival reflected greater disease severity. A multivariable model was then used to assess whether differences in quality-of-life trajectories by place of residence persisted after adjustment for survival time; predicted trajectories are presented graphically.

Survival analysis was conducted using a Kaplan–Meier approach for all participants with available enrollment and exit dates. All analyses and visualizations were performed using Stata/MP version 14.0 (StataCorp, College Station, TX, USA). Confidence intervals are reported to convey the precision of estimates.

## 3. Results

From the total of 183 patients admitted to the Palliative Care Service during the study period, 116 had an oncologic diagnosis and completed at least one PedsQL 3 Cancer Module, and 166 families completed at least one PedsQL Family Impact Module questionnaire. Patient characteristics and baseline scores are reported in Table 1.

At baseline, the mean score of all 116 oncologic patients was 58.9. At three and six months, the score increased to 68.4 and 77.9, respectively (*p* < 0.001), with significant improvements observed across all subscales. The domains showing the most significant improvement were pain, procedural anxiety, and communication (Table 1 and Table 2, and Figure S3).

Participants in the lower socioeconomic strata (AMAI categories D and D+) tended to have statistically nonsignificant lower baseline PedsQL Cancer Module scores than those in higher AMAI categories (mean difference: −6.32, 95% CI: −15.9 to 3.2). No significant associations were observed between baseline PedsQL Cancer Module scores and sex, age group, neoplasm type, place of residence, or survival time (Table S3).

**Figure 1:**
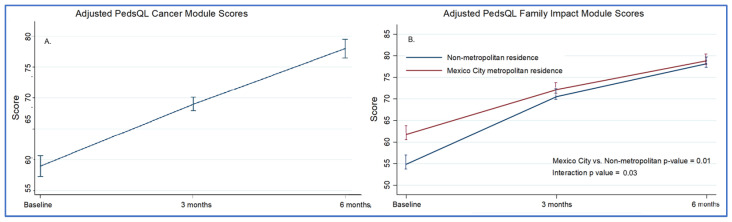
(**A**) PedsQL Cancer Module scores over time, adjusted for age. (**B**) PedsQL Family Impact Module scores over time by place of residence, adjusted for age, oncologic diagnosis, and survival < 12 months. Bars represent standard errors. Full models are presented in Tables S2 and S4.

Caregivers of 166 children completed the PedsQL™ 2.0 Family Impact Module. Total scores improved from 60.1 to 78.8 over six months (*p* < 0.001), with significant gains in the emotional, cognitive, and family relationship domains (Table S2, Figure S4). Families of children in the age group of 5–7 years had lower scores over time (adjusted difference: −8.1, 95% CI: −15.9 to −3.8), as did those with an oncologic diagnosis (adjusted difference: −13.0, 95% CI: −19.6 to −6.3) and families living outside the Mexico City metropolitan area (adjusted difference: −8.6, 95% CI: −14.7 to −2.6). There was a significant interaction between time and location of residence, indicating a higher rate of improvement among families living in non-metropolitan areas (Figure 1, Table S3). These associations persisted after multivariable analysis (Table S4).

**Figure 2:**
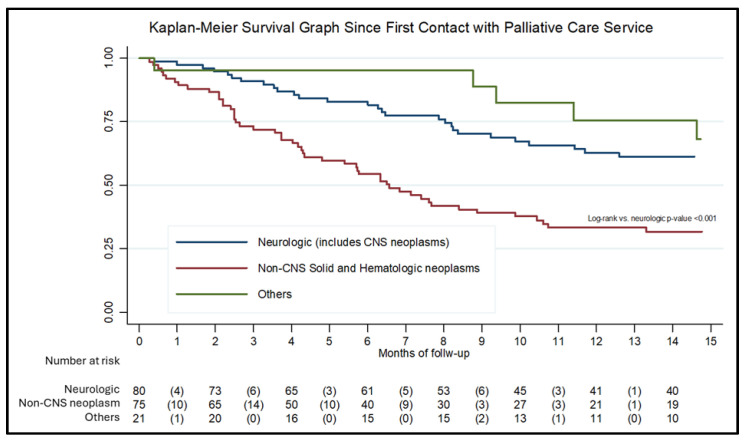
Kaplan–Meier survival function by diagnostic classification.

For the survival analysis, 176 records with complete date information were included. Figure 2 presents the Kaplan–Meier survival curve. Median survival was 11.4 months for the overall cohort, 6.5 months for patients with non-CNS solid or hematologic neoplasms, and 29 months for patients with neurologic conditions, including CNS neoplasms. Most patients (68.7%) died at home, while the remainder died in the hospital. No significant associations were observed between diagnosis, place of residence, or socioeconomic status and the place of death.

## 4. Discussion

This study describes quality-of-life trajectories following enrollment in a structured pediatric palliative care program in a middle-income country. Overall, we observed improvements in the reported quality of life among children and their families over time. These findings are consistent with global data showing that early palliative interventions are associated with improved symptom management and reduced psychosocial burden, particularly when care is delivered through multidisciplinary, family-centered models.

Consistent with previous reports from high-income settings, our results align with those of Currow et al., who documented improvements in symptom control and patient-reported outcomes following structured, patient-centered palliative care programs [17]. Andriastuti et al. observed clinically meaningful improvements only in the home-based pediatric palliative care intervention group, with effect sizes comparable to those observed in our study [8]. Taken together, these international findings underscore the importance of integrating pediatric palliative care services early in the disease course and tailoring them to the needs of both patients and their families, particularly in resource-constrained settings. However, direct comparisons should be interpreted cautiously, given differences in study design and health system contexts.

One of the most relevant findings of this study was the difference in quality of life improvement trajectories between families living within the Mexico City metropolitan area and those living farther away. Residence was used as a proxy for the level and modality of engagement with the palliative care program. Based on this assumption, our initial hypothesis was that families living farther from the hospital would experience smaller improvements in quality of life than metropolitan residents. Unexpectedly, despite having lower baseline quality-of-life scores, these families demonstrated greater improvement over time and reached levels comparable to those of local families. This difference in trajectories persisted after adjustment for survival time, which served as a proxy for clinical status, suggesting that the observed improvement cannot be explained solely by baseline differences or selective mortality. One possible explanation is a reduction in travel burden and hospital visits after enrollment, along with increased reliance on coordinated care, telephone follow-up, and home-based support. Together, these findings support efforts toward the decentralization of pediatric palliative care services and highlight the potential role of telehealth and community-based outreach in improving access for families living far from tertiary care centers. Similar multidisciplinary, community-oriented strategies have shown promising results in other low- and middle-income settings [18,19].

Families of children with non-oncologic conditions often face prolonged caregiving demands without clear prognostic timelines. Pediatric palliative care programs should adapt their models to provide sustained, longitudinal support for such populations beyond the typical oncology-based frameworks.

Our data highlight prolonged survival among a subset of patients, particularly those with neurological disorders. This pattern has important implications for the planning of pediatric palliative care services as it reflects a growing burden of care associated with long-surviving, non-oncologic, and severely disabled patients who require sustained multidisciplinary support over an indefinite period. These findings underscore the need for health systems to recognize and adequately resource pediatric palliative care services not only for terminal oncologic care but also for the growing population of children living with life-limiting conditions of diverse and often prolonged trajectories.

Several limitations should be acknowledged. First, this study’s single-arm design precludes causal inference; for ethical reasons, it is not permissible in our setting to include a group of eligible children who do not receive palliative care once referred. Second, although overall attrition was substantial, it was driven predominantly by mortality, which is inherent to pediatric palliative care populations. Third, the predominance of oncologic diagnoses may limit generalizability to non-cancer populations, although the inclusion of neurological and other conditions provides valuable insight into heterogeneous trajectories of care. Although disease-specific PedsQL modules could provide additional insight into symptom burden among children with non-oncologic conditions, their use was not feasible in this study. The PedsQL Cancer Module was therefore restricted to oncologic patients, while the PedsQL Family Impact Module was applied uniformly to families of both oncologic and non-oncologic patients, reflecting the program’s family-centered approach. These findings underscore the importance of evaluating family outcomes across diagnostic categories and highlight the need for future funding and research to support the use of appropriate quality-of-life instruments in non-oncologic pediatric palliative care.

Despite these limitations, this study provides meaningful hypothesis-generating evidence. It raises important questions for future research, particularly regarding which components of pediatric palliative care are most influential in improving quality of life for families of children with severe illnesses. Our findings suggest that enabling families to care for their children at home—without the need for frequent travel to a distant tertiary-care hospital—may be a key contributing factor.

The study also has notable strengths, including a high follow-up rate among surviving participants, the use of validated Spanish-language instruments, and robust longitudinal modeling of quality-of-life trajectories.

In a middle-income setting such as Mexico, where access to specialized pediatric palliative care varies widely by geography, these findings underscore the potential impact of structured programs in mitigating factors that would otherwise limit quality-of-life improvement, particularly for families living outside metropolitan areas. From a policy perspective, our findings highlight the need for health systems to recognize pediatric palliative care not only as end-of-life oncologic care, but also as a longitudinal service for children with diverse, often prolonged, life-limiting conditions. Expanding access to multidisciplinary palliative care, particularly for families living outside metropolitan areas, may help reduce inequities and improve quality of life for both children and their caregivers. Future research should focus on identifying modifiable components of palliative care programs that best explain observed improvements in quality of life, as well as on evaluating the economic impact of such programs from families’ perspectives.

## 5. Conclusions

Pediatric palliative care services were associated with favorable trajectories of quality of life among children with life-limiting conditions and their families, especially in those residing outside the hospital’s metropolitan area. These findings highlight the importance of ensuring sustained and equitable access to pediatric palliative care, regardless of diagnosis, geographic location, or socioeconomic background.

## Figures and Tables

**Table 1:** Characteristics of included participants at baseline.

	PedsQL Cancer Module Scores (N = 116)		PedsQL Family Impact Module Scores (N = 166)	
	n (%)	Mean (SD)	n (%)	Mean (SD)
Age at first contact ^1^				
0–1	-	-	12 (7.2%)	64.6 (18.0)
2–4 years	23 (19.8%)	59.2 (16.5)	36 (21.7%)	59.5 (21.4)
5–7 years	30 (25.9%)	53.2 (18.8)	35 (21.1%)	52.1 (18.2)
8–12 years	31 (26.7%)	60.6 (18.8)	37 (22.3%)	60.9 (16.5)
13–18 years	32 (27.6%)	62.2 (19.0)	46 (27.7%)	65.3 (17.4)
Sex				
Male	48 (41.4%)	59.1 (17.9)	72 (43.4%)	61.0 (17.8)
Female	68 (58.6%)	59.4 (20.2)	94 (56.6%)	59.0 (20.1)
Underlying medical condition				
Oncologic		59.2 (18.8)		57.5 (18.1)
CNS Neoplasia	52 (44.8%)	60.2 (19.4)	62 (37.4%)	58.3 (18.4)
Solid Neoplasm	40 (34.5%)	56.4 (19.3)	43 (25.9%)	56.6 (20.5)
Hematologic Neoplasm	24 (20.7%)	61.7 (16.9)	26 (15.7%)	56.9 (13.3)
Neurologic		-	17 (10.2%)	69.5 (17.1)
Heart disease		-	7 (4.2%)	68.5 (14.4)
Other		-	11 (6.6%)	73.6 (15.1)
Residency				
Mexico City and Metropolitan Area	68 (60.7%)	59.5 (20.1)	60 (36.2%)	64.2 (17.7)
Non-metropolitan	44 (39.3%)	58.7 (17.3)	106 (63.8%)	53.5 (18.2)
AMAI socioeconomic level ^2^ (n = 69)				
D	8 (12.9%)	48.3 (19.0)	8 (11.6%)	54.4 (15.1)
D+	37 (59.7%)	52.1 (15.1)	42 (60.9%)	56.9 (17.1)
C−	10 (16.3%)	59.9 (19.1)	10 (14.5%)	56.9 (18.4)
CM	4 (6.5%)	64.3 (20.5)	5 (7.2%)	60.4 (22.0)
C+	2 (3.2%)	55.1 (43.8)	3 (4.4%)	74.3 (19.1)
AB	1 (1.6%)	36.5 (-)	1 (1.5%)	42.4 (-)
Survival time				
<3 months	22 (19%)	60.9 (18.9)	26 (16%)	58.7 (21.2)
3–6 months	18 (16%)	63.1 (18.0)	20 (12%)	56.6 (14.4)
6–9 months	17 (15%)	57.8 (16.3)	21 (13%)	59.2 (15.9)
9–12 months	7 (6%)	55.6 (21.8)	12 (7%)	53.6 (17.6)
>12 months	44 (38%)	58.2 (18.1)	69 (42%)	62.6 (18.4)
Lost to follow-up < 12 months	8 (7%)	52.4 (24.8)	18 (11%)	61.8 (24.1)

^1^ Age stratification was performed according to age groups defined by the PedsQL versions. ^2^ Socioeconomic level according to the AMAI.

**Table 2:** PedsQL^TM^ 3.0 Cancer Module subscales over time.

	Baseline n = 116 ^1^		3 Months n = 98		6 Months n = 79		
	Mean	SD	Mean	SD	Mean	SD	*p* ^2^
PedsQL-total	58.9	18.5	68.4	14.6	77.9	15.9	<0.001
PedsQL-pain and hurt	59.0	29.0	67.4	24.8	83.2	19.1	<0.001
PedsQL-nausea	69.1	23.3	76.2	19.9	83.8	19.6	<0.001
PedsQL-procedural anxiety	45.3	34.3	57.2	30.6	73.2	33.4	<0.001
PedsQL-treatment anxiety	56.2	33.7	70.6	27.4	78.5	24.5	<0.001
PedsQL-worry	43.6	29.2	50.4	25.4	60.9	29.9	<0.001
PedsQL-cognitive problems	57.9	25.4	64.9	23.5	74.3	23.2	<0.001
PedsQL-communication	55.2	34.1	80.9	23.8	90.1	19.0	<0.001
PedsQL-perceived physical appearance	75.1	28.3	76.7	22.4	84.2	21.1	0.027

^1^ Participants with an oncologic diagnosis and older than two years old. ^2^ *p*-value, repeated measures ANOVA.

## Data Availability

The original contributions presented in this study are included in the article/Supplementary Material. Further inquiries can be directed to the corresponding author.

## Supplementary Materials

The following supporting information can be downloaded at https://www.mdpi.com/article/10.3390/children13020196/s1, Table S1: PedsQL^TM^ 2.0 Family Impact Module over time; Table S2: Regression models for PedsQL Cancer Module (N = 126); Table S3. Regression models for PedsQL^TM^ 2.0 Family Impact Module score (N = 116); Table S4: Multivariate model^1^ for PedsQL^TM^ 2.0 Family Impact Module; Figure S1: Participant flow diagram for quality-of-life assessment in oncologic patients. Figure S2: Participant flow diagram for family impact assessment. Figure S3: PedsQL Cancer Module subscales over 6 months; Figure S4: PedsQL Family Impact subscales over 6 months.

## Permission for the Use of Instruments

PedsQL™, Copyright © 1998 JW Varni, Ph.D. All rights reserved. Request #135535. For permission and access: Mapi Research Trust, Lyon, France.

## Abbreviations

The following abbreviations are used in this manuscript:

AMAI	Mexican Association of Market and Opinion Intelligence
CNS	Central nervous system
