# Supplementary material for: Improved Quality of Life in Children and Families Following Enrollment in a Pediatric Palliative Care Program: A Prospective Cohort Study
Source: Children (Basel). 2026 Jan 30;13(2):196. doi: 10.3390/children13020196 (PMC12939939; doi:10.3390/children13020196)
Supplement: Supplementary file 1 [file children-13-00196-s001.zip › children-4049065-supplementary.pdf]

children

Supplementary Materials

Table S1. PedsQL™ 2.0 Family Impact Module over time.

	**Basal n= 166[1]**		**3 months n= 100**		**6 months n= 82**		***p*-value[2]**
	**Mean**	**SD**	**Mean**	**SD**	**Mean**	**SD**	
PedsQL-family total	60.1	18.7	70.9	12.8	78.8	10.7	<0.001
PedsQL-family HRQL	63.4	19.9	74.1	14.1	81.0	12.4	<0.001
PedsQL-family functioning	67.8	24.1	81.1	16.6	88.8	12.1	<0.001
Physical functioning	64.9	22.1	76.3	15.7	81.7	15.5	<0.001
Emotional functioning	50.9	28.1	63.9	24.2	71.8	22.6	<0.001
Social functioning	64.9	26.2	76.4	19.8	84.0	13.6	<0.001
Cognitive	72.9	23.9	80.0	17.9	87.0	14.3	<0.001
Communication	63.1	26.8	73.3	21.4	85.3	13.3	<0.001
Worry	33.0	25.9	40.1	26.7	50.1	24.8	<0.001
Daily activities	61.2	28.3	75.3	20.3	83.8	16.7	<0.001
Family relationships	72.2	26.1	85.1	18.1	91.8	14.0	<0.001

[1]All diagnoses included. [2] Repeated measures ANOVA. HRQL: health-related quality of life.

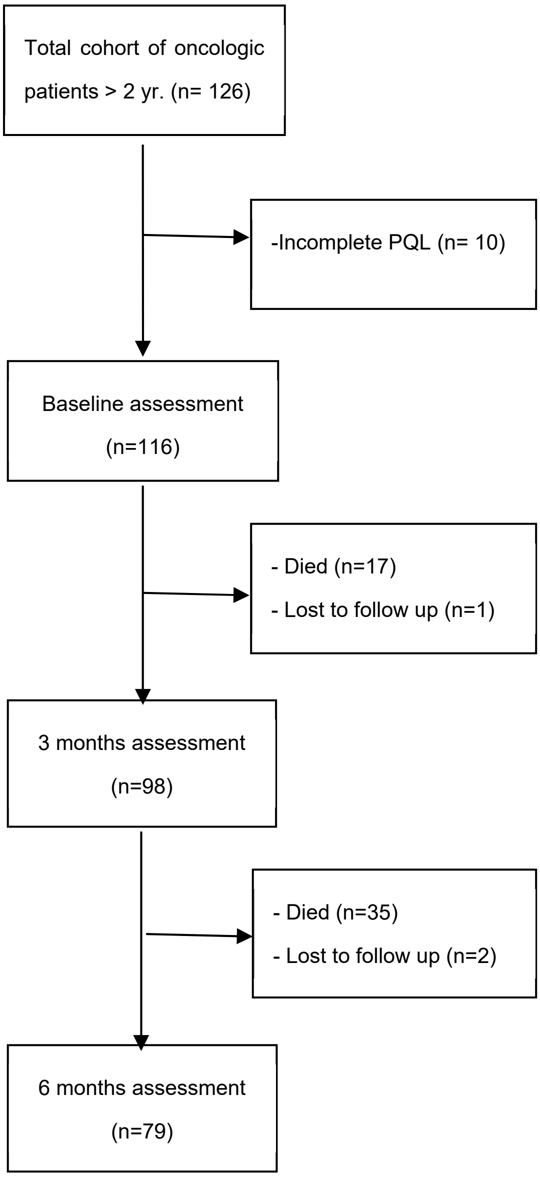

Figure S1. Participant flow diagram for quality-of-life assessment in oncologic patients.

Table S2. Regression models for PedsQL Cancer Module (N=126).

	**Factors Baseline Assessment**[1]			**Factors Adjusted for Time**[2]			**Time-Factor Interaction**[2]		
**Model**	**β**	**95% CI**		**β**	**95% CI**		**β**	**95% CI**	
**1. Time**									
Time: 3 months	**-**			**9.99**	**6.57**	**13.41**	-		
Time: 6				**18.92**	**13.76**	**24.08**			
**2. Age at first contact + time (Ref. 5-7 y)**									
Time: 3 months	-			**14.49**	**8.07**	**20.90**	-		
Time: 6 months	-			**24.28**	**14.59**	**33.97**	-		
2–4 years	2.97	-3.32	9.25	6.36	-3.29	16.00	-4.58	-14.58	5.42
8–12 years	0.94	-4.81	6.69	7.16	-1.79	16.11	**-12.64**	**-21.70**	**-3.58**
1318 years	5.38	-0.25	11.01	8.80	-0.08	17.67	-1.39	-10.26	7.48
**3. Sex + time (Ref. Female)**									
Time: 3 months	**-**			**7.51**	**2.05**	**12.97**	**-**		
Time: 6 months	**-**			**17.31**	**9.08**	**25.53**	**-**		
Male	-0.88	-7.82	6.07	-0.84	-7.56	5.89	3.99	-3.01	10.99
**4.Diagnosis + time (Ref. Solid Neoplasm)**									
Time: 3 months	**-**			**12.01**	**6.29**	**17.72**	**-**		
Time: 6 months	**-**			**16.41**	**7.90**	**24.92**	**-**		
Hematologic Neoplasm	5.37	-4.13	14.87	5.70	-3.48	14.87	-5.78	-15.18	3.62
CNS Neoplasm	3.10	-4.64	10.84	3.00	-4.48	10.48	-1.78	-9.39	5.83
**5.Zone + time (Ref. Mexico City Met.)**									
Time: 3 months	**-**			**8.39**	**4.01**	**12.78**	**-**		
Time: 6 months	**-**			**16.70**	**9.92**	**23.47**	**-**		
No-Metropolitan	-0.25	-7.43	6.93	-0.24	-7.23	6.74	3.92	-3.15	11.00
**6. Socioeconomic level + time (Ref. ≥C-)**									
Time: 3 months				**12.08**	**5.18**	**18.98**	**-**		
Time: 6 months				**24.41**	**13.25**	**35.56**	**-**		
Lower (AMAI levels D and D+)	-6.32	-15.85	3.21	-6.23	-15.03	2.58	1.59	-6.50	9.68
**7. Survival (Ref. > 12 months)**									
Time: 3 months				**12.40**	**7.25**	**17.55**			
Time: 6 months				**21.16**	**13.52**	**28.80**			
Survival < 6 months	3.61	-4.22	11.45	3.88	-3.67	11.42	-2.22	-10.11	5.66
Survival 6–12 months	-1.08	-10.18	8.03	-0.73	-9.48	8.03	**-9.19**	**-17.76**	**-0.61**

[1]Simple linear regressions. [2] Linear mixed-effects models; participant level was included as a random effect, and time (baseline, 3, and 6 months), factor, and their interaction were included as fixed effects. Bold values: $p < 0.05$

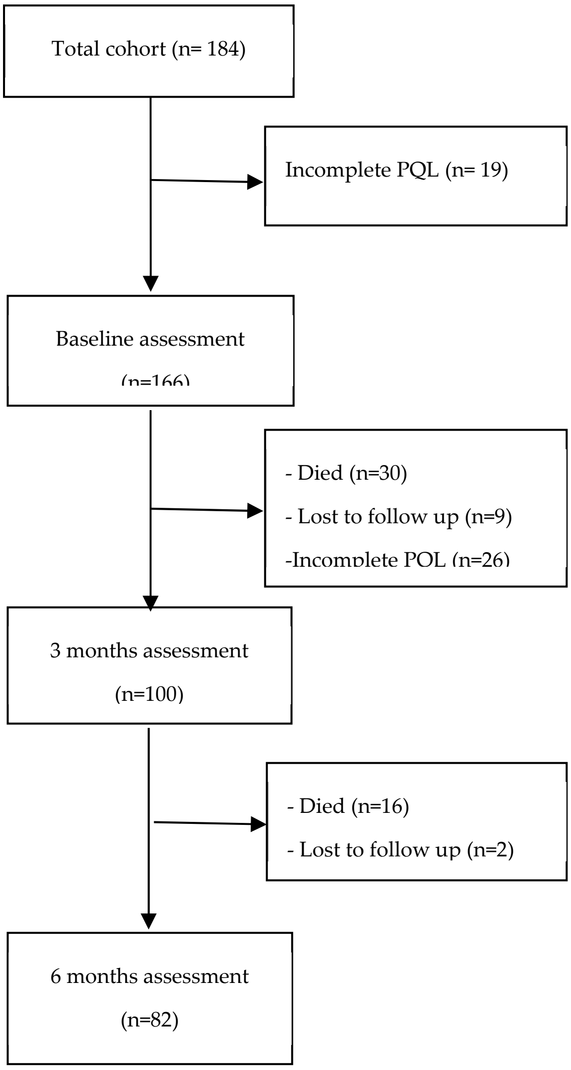

Figure S2. Participant flow diagram for family impact assessment.

Table S3. Regression models for PedsQL™ 2.0 Family Impact Module score (N=116).

Model	Factors Baseline Assessment[1]			Factors Adjusted for Time[2]			Time-Factor Interaction[2]		
	ß	95% CI		ß	95% CI		ß	95% CI	
**1.Time**									
Time: 3 months				**11.75**	**9.56**	**13.95**			
Time: 6 months				**18.74**	**15.39**	**22.08**			
**2.Age at first contact + time (Ref. 5-7 y)**									
Time: 3 months	-			**12.63**	**8.20**	**17.05**			
Time: 6 months	-			**21.99**	**15.20**	**28.78**			
0–2 years	8.13	-0.23	16.49	**12.56**	**0.59**	**24.53**	-1.91	-12.76	8.94
2–4 years	**5.76**	**0.19**	**11.32**	7.40	-0.79	15.59	2.67	-4.01	9.34
8–12 years	**7.65**	**2.44**	**12.87**	**8.92**	**0.76**	**17.08**	-1.16	-7.42	5.10
13–18 years	**9.42**	**4.41**	**14.44**	**13.22**	**5.49**	**20.95**	-2.93	-8.94	3.08
**3.Sex + time (Ref. Female)**									
Time: 3 months				**11.25**	**7.84**	**14.65**			
Time: 6 months				**17.12**	**11.95**	**22.30**			
Male	3.61	-0.08	7.30	2.13	-3.49	7.75	0.81	-3.64	5.27
**4.Diagnosis + time (Ref. Non-Oncologic)**									
Time: 3 months				6.16	-2.08	14.39			
Time: 6 months				-1.58	-22.23	19.06			
Oncologic	**-12.50**	**-19.30**	**-5.71**	**-12.97**	**-19.63**	**-6.31**	6.91	-1.63	15.4673
**5.Zone + time (Ref. Mexico City Met.) Metropolitan)**									
Time: 3 months				**10.07**	**7.08**	**13.06**			
Time: 6 months				**18.08**	**13.57**	**22.59**			
No-Metropolitan	**-10.59**	**-16.40**	**-4.79**	**-8.63**	**-14.72**	**-2.55**	**7.40**	**2.91**	**11.90**
**6.Socioeconomic level + time (Ref. ≥C-)**									
Time: 3 months				**12.58**	**7.25**	**17.91**			
Time: 6 months				**19.61**	**11.49**	**27.73**			
Lower (AMAI levels D and D+)	-1.62	-6.60	3.37	-3.44	-12.36	5.57	1.28	-4.81	7.38
**7. Survival time if lived (ref >12m)**									
Time: 3 months				**12.95**	**9.76**	**16.15**			
Time: 6 months				**18.80**	**13.96**	**23.64**			
Survived <6months	-4.79	-11.56	1.98	-4.22	-10.75	2.30	-0.74	-6.17	4.69
Survived 6–12 months	**-5.49**	**-13.02**	**2.04**	-5.49	-12.78	1.79	-5.19	-10.85	0.48

[1]Simple linear regressions. [2] Linear mixed-effects models; participant level was included as a random effect, and time (baseline, 3, and 6 months), factor, and their interaction were included as fixed effects. Bold values: $p < 0.05$.

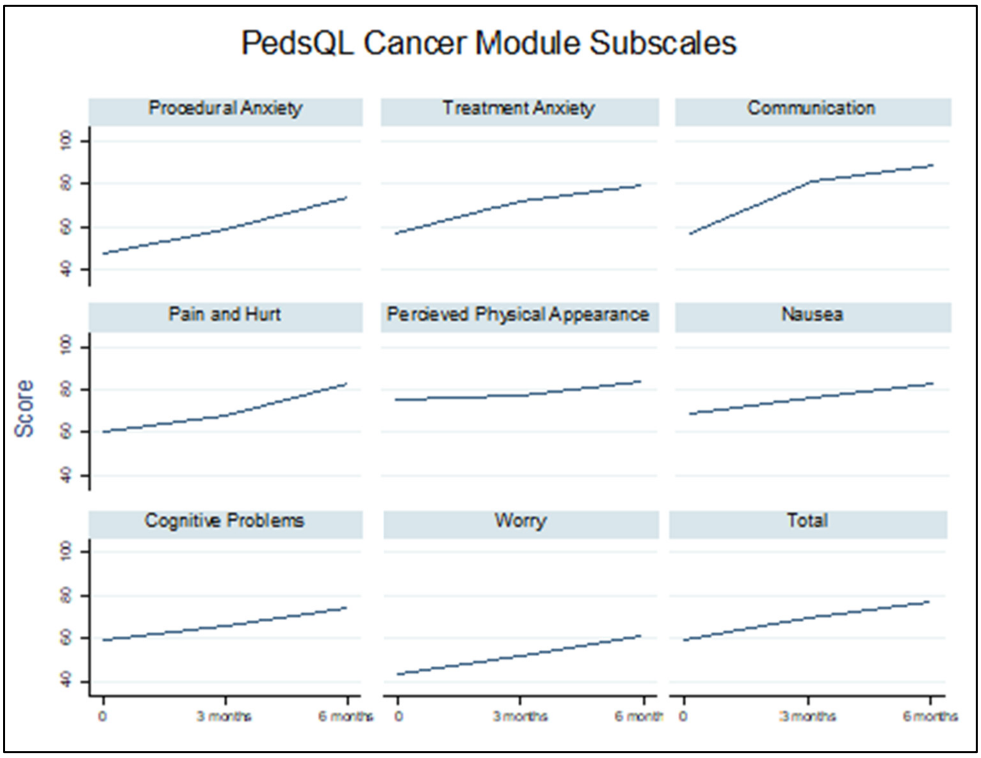

Figure S3. PedsQL™ 3.0 Cancer Module subscales over 6 months.

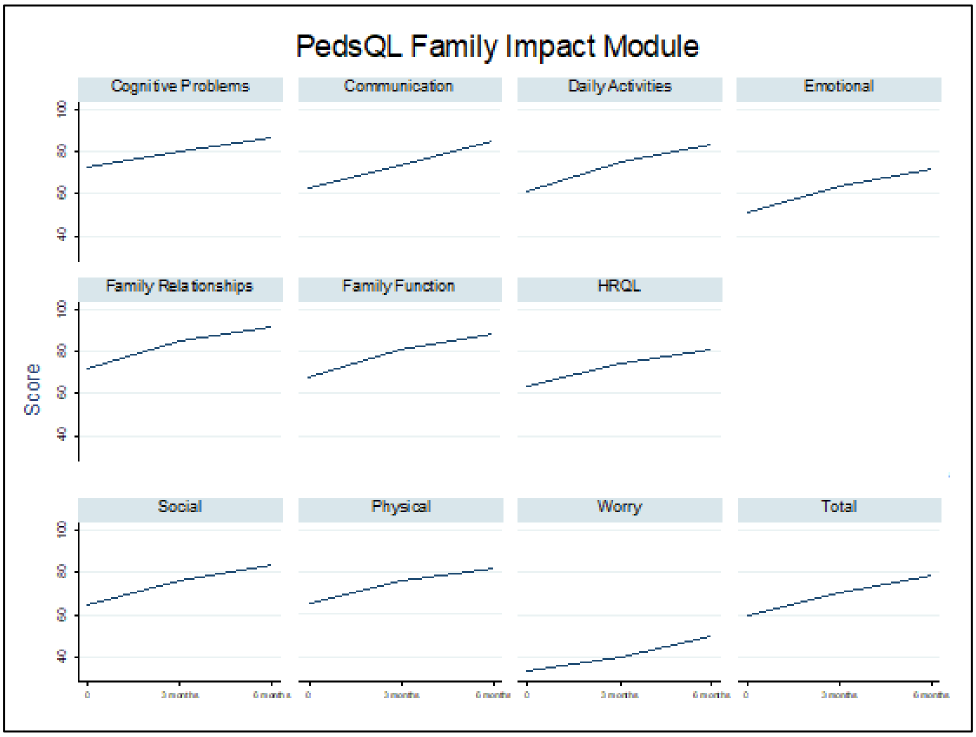

Figure S4. PedsQL™ Family Impact Module subscales over 6 months.

Table S4. Multivariate model[1] for PedsQL™ 2.0 Family Impact Module.

	β	*p*-value	95% CI	
**Time**				
Time: 3 months	**13.22**	**<0.001**	**9.67**	**16.76**
Time: 6 months	**20.56**	**<0.001**	**15.17**	**25.94**
**Age**				
5–7 years old	**-8.09**	**<0.001**	**-15.90**	**-3.77**
**Diagnosis (Ref. Non-oncologic)**				
Oncologic	**-9.84**	**0.001**	**-15.90**	**-3.77**
**Zone + time (Ref. Mexico City Met.)**				
No-Metropolitan	**-6.91**	**0.01**	**-12.38**	**-1.44**
No-Metropolitan x time	**5.25**	**0.03**	**0.55**	**9.96**
**Survival (Ref: ≥ 12 months)**				
Survival <12 months x time	**-6.23**	**0.004**	**-10.51**	**-1.95**

[1]Linear mixed-effects models; participant level was included as a random effect, and time, age 5–7 years, oncologic diagnosis, zone of residence, zone of residence x time, and survival <12 months x time were included as fixed effects.
